# Examining the divergent effects of perceived inclusion of ethnic minorities on majority and minority groups’ inter-ethnic responses

**DOI:** 10.3389/fpsyg.2023.1242595

**Published:** 2023-11-08

**Authors:** Iris Andriessen, Seval Gündemir, Joost W. S. Kappelhof, Astrid C. Homan

**Affiliations:** ^1^Department of Pedagogy, Fontys University of Applied Science, Eindhoven, Netherlands; ^2^Department of Work and Organizational Psychology, University of Amsterdam, Amsterdam, Netherlands; ^3^The Netherlands Institute for Social Research (SCP), The Hague, Netherlands

**Keywords:** diversity and inclusion, ethnocentrism, interethnic contact, paradoxes of perceived inclusion, minority - majority

## Abstract

This study examines the paradoxical effects of a perceived inclusive environment for ethnic minorities. We argue that while perceptions of an inclusive environment may be associated with more positive intergroup attitudes and affect among minority groups, they may instill a sense of threat among the majority group, resulting in negative intergroup sentiments and attitudes towards minorities. We analyzed data from two waves of a nationally representative survey conducted in the Netherlands (*n*_total_ = 11,897) comprising minority and majority groups. We find support for the proposed paradoxical relationship between the perceived inclusionary climate towards minorities and the attitudes of the majority and minority groups. The results indicate that when perceiving the national climate to be more inclusive towards minorities, the majority group tends to report higher levels of ethnocentrism, avoid direct inter-ethnic contact, and oppose ethnic diversity in general. Among minority groups, a perceived inclusive climate is linked to lower levels of ethnocentrism and a higher willingness to engage in inter-ethnic interactions with the majority group. The results unexpectedly also show that the perception of an inclusionary climate is positively related to opposition to increased ethnic diversity among minority groups. We discuss theoretical and societal implications, while also considering the contextual relevance and limitations of our approach.

## Introduction

“*I hope he gets rewarded for his hard work. That he gets the same chances as the minorities who have come to live here*.” – translated quote from a grandmother talking about her grandson (Elsevier Weekblad, 2016).

Ethnic-cultural diversity in Europe has rapidly increased in the past decades. In 2022, nearly 24 million people living in the EU were citizens of non-member countries (Eurostat, 2022). Similarly, in the Netherlands the percentage of people with a migration background, i.e., individuals who themselves or have at least one parent born abroad (as defined by Statistics Netherlands [CBS], the Dutch national statistics institute in 2016), is on the rise with 20% in 2010, to 22% in 2015, 24% in 2020 and 25% in 2022.^1^ This large and ever-growing minority population still faces many challenges, ranging from discrimination in social and work settings (Vogt, 2005; Andriessen et al., 2010; Wrench, 2017) to higher levels of unemployment (Huijnk, 2016; Galvin, 2017). There has also been an observable increase in ethnic minorities expressing their concerns about the injustices they have experienced through various social movements. Hence many governments and other collectives engage in initiatives to improve the societal standing of minorities, focusing on, for example, how these groups can become part of the social structures, can be respected, and be fairly treated (Ward et al., 2018). Altogether, these efforts are directed at the social inclusion of ethnic minority groups, with the ultimate goal of promoting social cohesion through favorable interethnic attitudes and contact.

At the same time, populist political narratives have increasingly emerged in both Europe and the United States. In this rhetoric, support for and affiliation with diversity by different groups (e.g., political parties, governments) is presented as a cultural and economic threat to the nation (Davidov et al., 2020). Political parties and movements relying on this narrative have emerged and received a substantial share of the votes and political power (Green et al., 2020). For example, the ‘leave’ campaign of the Brexit referendum drew heavily on the supposed threats of immigration for the United Kingdom (Visintin et al., 2018). The belief that the majority group will eventually become a minority and as a consequence lose power over the country’s future has fueled feelings of threat and consequently support for political parties that oppose migration and minority inclusion (Kešić and Duyvendak, 2019).

Drawing on instrumental models of group conflict (Quillian, 1995; Esses et al., 1998; Scheepers et al., 2002; Meuleman et al., 2009), here we further examine this bifurcation. This model considers perceived competition for resources as an important determinant of intergroup attitudes and behavior. The competition may be about economic or material resources, such as money, jobs and housing. However, research suggests that perceived cultural threat, such as an anticipated clash of values or fear of losing a particular cultural identity, may be as important (Ivarsflaten, 2005; Sniderman and Hagendoorn, 2007; Schneider, 2008).

A perceived national climate that promotes equal rights, fair treatment, and respect for minorities may trigger a sense of threat in the majority group. Such threat arises from the perceived or anticipated competition between minorities and the majority group for limited resources (e.g., jobs, housing), and for symbolic or cultural hegemony, as minories can be seen to challenge the cultural predominance that the majority group wishes to maintain (Allport, 1954; Stephan and Renfro, 2002; McLaren, 2003; Semyonov et al., 2008). Intriguingly, such perceptions or anticipations may not accurately reflect actual societal changes (Peyton et al., 2022).

When intergroup relationships are perceived through a zero-sum competition lens, a gain for minorities is perceived as a loss for the majority (Esses et al., 1998, 2005). This can exacerbate feelings of resentment, fear, and hostility towards minority groups among the majority population. Among minority group members, however, an inclusive national climate may evoke fundamentally different responses. Minorities perceiving an inclusive national climate feel more accepted and valued by the larger society which in turn would enhance their sense of belonging and well being. As a result, they may perceive and seek positive interactions with members of the majority group and view diversity as an asset to society. When both sides of the coin are taken into consideration, it suggests that a national inclusion climate may create a context in which one group’s approach intentions may be met with avoidance from the other, resulting in strained intergroup relationships and interactions.

We test and replicate the proposed paradoxical effects of perceived inclusion focusing on the ethnic majority and minority groups in the Netherlands. We use data coming from two waves - collected with a four-year gap - of a repeated cross-sectional survey called the Dutch Survey on the Integration of Minorities (SIM). In both waves data was collected among a representative sample of each of the four largest non-western minority groups in the Netherlands: individuals with a Turkish, Moroccan, Surinamese or Dutch-Carribean migration background (as well as a comparative group of persons without a migration background; the majority group).^2^ Together, these four minority groups make up almost 60% of the total population with a non-western migrant background and more than 30% of the total immigrant population in the Netherlands. The survey taps into a broad range of subjects and is unique because of the large and representative sample of different minorities, reaching not only the highly educated minorities, but also those with lower levels of formal education, less or no Dutch language proficiency, or those from lower income groups by (also) using face-to-face interviews conducted by interviewers in different languages.

This work makes critical contributions by testing and extending the propositions of the instrumental model of group conflict (Quillian, 1995; Esses et al., 1998). For example, the main premise of this theory is that intergroup conflict stems from the perception of competition for access to valuable resources. We argue that signals of such competitive contexts can be construed differently by members of groups from higher versus lower status positions, who have a greater or smaller share to start with. Consequently, their responses may differ as a factor of these initial group status differences. Given that the majority of existing research has focused on investigating responses to such contexts from the perspectives of the majority, high status groups (Schlueter et al., 2013; Green et al., 2020), our understanding of how such contexts affect minority and lower-status groups remains relatively underdeveloped. This work contributes to theory building from minority groups’ point of view and tests the applicability of the instrumental model of group conflict to groups with lower social standing. In so doing, it adds an additional theoretical layer to the model and extends its reach. Second, the instrumental model of group conflict suggests that perceived social mobility can elucidate a sense of threat in the majority group which prompts them to want to reduce the source of competition through -among other things- negative attitudes and avoidant tendencies. We gauge the validity of this argument in the context of the Netherlands, unraveling some of the unique forms such negative responses can take. Third, the proposed paradoxical effects of perceived national inclusion climate could explain, at least to some extent, why creating inclusionary climates may not always have the intended positive effects on intergroup contact and can even create unintended negative effects. These findings provide valuable insights for policymakers when designing interventions aimed at promoting minority inclusion. It is important to consider the potential unintended consequences of such interventions to ensure that their benefits are not negated by unforeseen outcomes. By taking into account the complexity of intergroup dynamics and the different perspectives of both majority and minority groups, policymakers can design more effective and sustainable interventions that promote positive intergroup relations.

## Theory and hypotheses

Increased migration has led many societies to seek ways to optimally include and enhance participation among minority and immigrant communities, through integrative and multiculturalist policies and efforts (Castles, 1992; Gryzmala-Kazlowska and Philimore, 2017). Notwithstanding whether these policies have, in fact, created the intended objective – that is, structural status gain among minority groups – they have contributed to the emergence of a social mobility narrative which contends that being part of traditionally disadvantaged ethno-racial groups no longer presents a hindrance to one’s potential success or accomplishments (Lum, 2009; Bobo, 2011). From the majority’s perspective a zero-sum view on social standing appears to be present: if minority groups are thought to encounter no realistic barriers for progress, perceptions of an environment allowing progress should result in these groups claiming more of the valued, yet limited resources (e.g., jobs), leaving less resources available for the majority group. Thus, in a world of finite resources, perceptions of societal contexts that allow or promote social mobility, such as those signaling opportunity-rich egalitarian environments for minority groups, can contribute to perceived competition among groups (Allport, 1954; Esses et al., 2005).

The instrumental model of group conflict suggests that resource stress (i.e., perceived limited access to valued resources by one’s group) and salient competitor outgroups (e.g., dissimilar groups in appearance or behavior) cause perception of competition and attempts to remove the source of competition (Esses et al., 1998, 2005). Perceived social mobility and opportunity in society can thus lead high status groups to experience “resource stress” because these signal (potential) changes in the societal hierarchy. The underlying idea is that environmental cues prompting changes to hierarchy can be threatening, as one’s own group may no longer have disproportional power and status.

Previous research has suggested that inclusionary environments can represent such cues as they constitute complex resource negotiation settings between groups (Eibach and Keegan, 2006). These settings make parties especially vigilant to (potential) gains and losses, creating fixed-pie perceptions of outcomes where others’ gain is perceived as one’s loss (De Dreu et al., 2000; Bazerman et al., 2001). Consequently, societal contexts aiming at inclusion, such as those enhancing egalitarian treatment and social standing of minority groups, can be perceived as a loss by the majority group members, who traditionally enjoy a more privileged societal position. That is, perceptions of losing their dominant position inflates perceptions of minorities’ progress (Eibach and Keegan, 2006). The opening quote from a 2016 article in a popular Dutch weekly journal also illustrates this, showing that, from a majority group member’s perspective, the chances minorities get may come at the expense of the majority group’s chances. Empirical research supports this idea. Evidence from a study shows that in the United States, white people’s perceptions of decreased bias towards African Americans are associated with increased perceptions of an anti-white bias, such that white participants perceive their own group to face more bias than African Americans (Norton and Sommers, 2011). More recent work demonstrates that the majority group members report a sense of threat primarily as a consequence of perceived (rather than actual) ethno-racial diversity, which they construe as competition for valued resources (Gorodzeisky and Semyonov, 2020).

This perceived threat can have significant impact on intergroup attitudes and affect, particularly towards minority groups. The instrumental model of group conflict suggests that individuals may employ various coping mechanisms to address perceived competition, such as expressing negative attitudes or making unfavorable attributions towards outgroups, as well as avoiding physical proximity to those groups (Esses et al., 1998, 2005). In addition, the improving position of minority groups may prompt a shift in political preferences towards conservatism, which reinforces the status quo (Craig and Richeson, 2018). Furthermore, meta-analytical evidence suggests that intergroup threat can lead to negative outgroup attitudes, potentially contributing to the rise of anti-immigration political movements (Boomgaarden and Vliegenthart, 2007).

### Majority group’s intergroup responses to inclusionary climate for minorities

The above discussion suggests that a perceived inclusionary climate can instigate negative responses of majority group towards minorities. We examine these responses in three areas: (a) negative intergroup affect, (b) overall negative attitudes towards diversity, and (c) avoidant behavioral tendencies in interethnic context. By examining these three areas, we provide critical insights that span a spectrum of possible intergroup reactions, with important implications for both theory and policy making.

Negative intergroup affect is most widely captured through ethnocentrism – generalized negative affect towards outgroups (Triandis, 1990). The perception of losing a privileged position in society can create a sense of threat to the interests of the majority group, leading to negative affect towards threatening outgroups (Smith, 1993). For example, when a group feels threatened, there can be an increase in out-group bias (Brown and Ross, 1982) and in-group favoritism (Breakwell, 1978). Therefore, we propose that the perceived inclusion of minority groups may correlate with heightened ethnocentrism among the majority group.

In addition to outgroup specific negative affect, we also investigate overall negative attitudes towards diversity manifested as resistance to diversity. This reflects an oppositional stance regarding the desirability and value of (increased) diversity for society (Velasco and Sansone, 2019). An inclusive society towards minorities is one that is open to increased minority representation. Increased numeric representation of minorities could endanger the majority group, since larger groups are believed to hold more power than smaller groups (Blumer, 1958). As a group grows in size, it can be perceived as more threatening since it has the potential to mobilize and advocate for a better position within society, especially in a democratic context. Hence, even the mere presence of cultural diversity in a neighborhood can be interpreted as a perceived threat to the majority group. Research has shown that there is a positive correlation between a sense of endangerment among the majority group and the overestimation of the perception of minority group size (Alba et al., 2005). Further, when under increased threat, the majority group is more likely to reject diversity and express negative attitudes towards minority groups (Outten et al., 2012; Danbold and Huo, 2015). Therefore, perceived minority inclusion may be associated with increased resistance to diversity among the majority group.

Finally, we investigate the avoidant tendencies exhibited by the majority group in response to an inclusive climate. We examine these tendencies in the context of the reluctance to engage in intergroup contact. We propose that perceived inclusion of minority groups is associated with increased reluctance to interethnic contact in the majority group. Unraveling the determinants of interethnic contact, under certain conditions, is crucial since contact can be a direct way to improve interethnic relations through more positive intergroup attitudes (Pettigrew et al., 2007), reduced threat (Green et al., 2020) and lowered prejudice towards minorities (Visintin et al., 2020). Intriguingly, the relationship between contact and intergroup attitudes has a reciprocal nature: contact can improve attitudes, and positive attitudes increase the likelihood of intergroup contact (Herek and Capitanio, 1996; Levin et al., 2003). Hence illuminating what drives contact (intentions) is key for understanding these unique dynamics of multi-ethnic societies. Taken together, this leads to the following prediction:

*Hypothesis 1a*: For the majority group, perceived climate of inclusion for minorities is positively associated with ethnocentrism, resistance to diversity, and reluctance to engage in interethnic contact with minority groups.

### Minority group’s intergroup responses to inclusionary climate for minorities

The instrumental model of group conflict mainly focuses on the intergroup dynamics resulting from perceived threats to the majority group’s social status or access to resources due to social mobility (Esses et al., 1998, 2005). It is important to consider the impact of these perceptions among minorities as well, since they can significantly affect their acculturation orientation (Bourhis et al., 1997). In fact, while climate for inclusion for minorities constitutes a potential threat to the majority group, for minorities it represents a welcome opportunity. For minorities (perceiving) social inclusion is undoubtedly beneficial. Numerous studies show that a sense of belonging and equitable treatment are associated with benefits among minority groups, including better school achievements, work and educational engagement, life satisfaction, and mental health (Walton and Cohen, 2011; Berry and Hou, 2016; Phalet and Baysu, 2020). Minority youth who experience more intergroup contact and less unequal treatment, report more belonging with the majority group (Kende et al., 2021). Hence, we contend that perceptions of an inclusionary climate should have opposite effects for minority groups compared to the majority group.

We anticipate that these divergent effects will manifest in intergroup affect and attitudes. The sense of belonging, respect, and fair treatment is associated with more positive attitudes towards the majority group, as it reduces anxiety around intergroup contact and concerns of rejection (Mendoza-Denton et al., 2002; Plant and Devine, 2003). This suggests a negative relationship between perceived climate for inclusion and ethnocentrism among minorities. Similarly, from the minority groups’ perspective, perceived inclusion climate for minorities may boost these groups’ pro-diversity attitudes. In general, minority groups are supportive of climates and initiatives aiming at improving the position of their own and other minority groups (e.g., Bobo and Hutchings, 1996; Harrison et al., 2006). Some research on neighborhood heterogeneity suggests that among minorities, increased presence of other groups lowers both prejudice towards and sense of competition with those other groups (Oliver and Wong, 2003). This suggests for minority groups that perceptions of an inclusion climate should be negatively associated with generalized resistance to diversity. Thus, we propose for minority groups reduced ethnocentrism and resistance to diversity in an environment perceived as inclusionary.

We also expect contrasting patterns when focusing on avoidant tendencies in the form of reluctance to engage in interethnic contact. Specifically, perceptions of an inclusionary climate should encourage minorities to seek contact with the majority group. Indeed, empirical studies have provided evidence that when minority groups perceive that their experiences and identity are recognized and valued by the majority, they tend to express greater intentions to engage in intergroup contact (Tropp and Bianchi, 2007). Building on this, we expect minorities’ approach- rather than avoidance-intentions to grow when they perceive the national climate to support their participation and mobility. This leads to the following prediction for the minority group:

*Hypothesis 1b*: For the minority group, perceived climate of inclusion for minorities is negatively associated with ethnocentrism, resistance to diversity, and reluctance to engage in interethnic contact with the majority group.

To test our model (see Supplementary Figure S1) we use two independent datasets comprising responses among several minority ethnic groups and a comparative (majority) group. Considering the cross-sectional nature of our data sets, it is important to note that our approach cannot establish causality or determine the direction of relationships. However, examining the model in two independent samples allows us to replicate the findings and demonstrate the stable (as opposed to incidental) nature of the patterns. Further, we focus on individual level subjective perceptions of a societal context. Consequently, our approach does not provide evidence concerning the accuracy or origins of these perceptions. We elaborate upon these limitations in the general discussion.

## Methods

### Survey and datasets

The data used for this study were collected as part of the Dutch Survey on the Integration of Minorities (SIM). This is a repeated, cross-sectional survey among Dutch citizens with no migration background (majority group) and the four largest, non-western minority ethnic groups living in the Netherlands (i.e., Dutch of Turkish, Moroccan, Surinamese, Dutch-Carribean descent). For the current study, we use data collected as part of the 2011^3^ and 2015 wave.^4^ The data that support the findings of this study are openly available in DANS at doi: 10.17026/dans-xfv-2vx4, and doi: 10.17026/dans-xep-by9x.

For each wave, Statistics Netherlands (CBS) drew samples from the national population register (inhabitants aged 15 and above). All sampled persons were invited to take part in the survey in a consent letter that was sent to their home address. The letter contained information about the research, how the data would be used and a statement about privacy. It also contained a free telephone number and email address for subjects with further questions on the research. Respondents received 15 euros for their participation (for more details on the SIM survey designs, see Andriessen and Kappelhof, 2015; Kappelhof, 2015). For reasons of brevity, we will refer to the groups in our study as Dutch, Moroccan, Turkish, Dutch-Carribean and Surinamese. The net samples of respondents used in the current study are included in Table 1.

**Table 1:** Sample characteristics.

Characteristic	SIM 2011	SIM 2015	Combined
Age, M (SD)	40.1 (16.9)	40.8 (17.2)	40.3 (17.0)
Gender (*n*)			
Male	3,301	2,359	5,660
Female	3,522	2,715	6,237
Group (*n*)			
Dutch-descent	1,395	1,046	2,441
Moroccan-descent	1,385	951	2,336
Turkish-descent	1,348	920	2,268
Dutch-Carribean descent	1,400	1,112	2,512
Surinamese-descent	1,295	1,045	2,340
Total	6,823	5,074	11,897

### Measures

CBS determines *ethnic group membership* by attending to one’s country of birth and the country of birth of their parents. Persons born in the Netherlands with both parents born in the Netherlands are classified as Dutch without a migration background. Persons who were either born themselves in a different country or had at least one parent born in a different country are classified as having a migration background (CBS, 2016).^5^ We dichotomized this variable to construct the variable (majority/minority group) for the purpose of our study distinguishing respondents without a migration background (“native Dutch”; majority group), and with a migration background (Turkish, Moroccan, Surinamese, or Dutch-Carribean background; minority group). This distinction was used to test for the differential effects of perceived climate for inclusion of minorities on the dependent variables in a multi group model (see analytic strategy).

The latent construct *Perceived climate for inclusion of minorities* was measured using four items that were scored on a 5-point scale (1 = completely disagree, 5 = completely agree). The items were “The Netherlands is a hospitable country for ethnic minorities” (hosp). “The Netherlands is open to foreign cultures” (open), “Ethnic minorities have every chance in the Netherlands” (chance) and “In the Netherlands the rights of ethnic minorities are respected” (rights).

*Ethnocentrism* was constructed using ‘feeling’ thermometers (Nelson, 2008). Each respondent rated all five groups (including their own ethnic group) on a scale ranging from 0 to 100, with 0 indicating very cold feelings towards that group and 100 indicating very warm feelings. For native Dutch participants ethnocentrism was calculated as the score on the native Dutch group (tempnatd) minus the mean score on the other four ethnic groups (temptur [Turkish background], tempmor [Moroccan background], tempsur [Surinamese background], tempant [Dutch-Carribean background]). For ethnic minorities we calculated ethnocentrism as the score on their respective ingroup (i.e., Turkish, Moroccan, Surinamese or Dutch-Carribean) minus their score for native Dutch (Valentino et al., 2013).

The latent construct *Resistance to diversity* was measured using three items with a 5-point scale (1 = completely disagree, 5 = completely agree). The items were: “Too many ethnic minorities live in the Netherlands” (many), “Neighbourhoods deteriorate when too many ethnic minorities live there” (deter), and “It is a good thing when a society consists of different cultures” (cultdiv). The last item was reverse coded, so that higher scores indicate higher levels of resistance to diversity.

The latent construct *Reluctance to engage in interethnic contact* was measured using two items with a 5-point scale (1 = not at all, 5 = very much). The items were adapted according to the ethnic background of the respondents and read: “How much would you object to one of your children having many friends from ethnic minority groups [native-Dutch participants]/ many native Dutch friends [ethnic minority participants]” (friends) and ‘How much would you object to one of your children choosing a partner from an ethnic minority group[native-Dutch participants]/ a native Dutch partner [ethnic minority participants]” (partner) (Johnson and Jacobson, 2005; Tropp et al., 2016). Higher scores indicate higher reluctance to engage in intergroup contact. Please see Supplementary Table S1 for an overview of the labels used in the present paper and those in the overarching data set.

### Analytical strategy

To test our hypotheses, we used a three-stage approach. In the first – preparatory – stage of the analyses, we performed a multi-group confirmatory factor analysis (MGCFA) to test for measurement equivalence of the latent concepts across minority and majority groups, using Mplus version 7.4 (Muthén and Muthén, 2011).^6^ This will be referred to as the measurement model (2011 M0 for 2011 and 2015 M0 for 2015).

After establishing measurement equivalence (i.e., that the constructs in the model measure the same thing for the different groups and therefore the estimates can be meaningfully compared), we used a multi group structural equation model. We focused on fitting the hypothesized structural relations between the different constructs for the minority and majority group.

Models 2011 M1 and 2015 M1 tested for the equivalence of the hypothesized relationships between the constructs associations between majority and minority groups, by constraining the structural paths between the constructs to be equal between majority and minority groups.^7^ Here we ask the question if the relationships between the constructs are equivalent for majority and minority groups in 2011 and 2015.

In a subsequent set of models, we released the restraints on the structural paths for the majority and minority groups to be equal for *perceived climate for inclusion of minorities* on the variables *ethnocentrism* (2011 M2a and 2015 M2a)*, reluctance to engage in interethnic contact* (2011 M2b and 2015 M2b) and *resistance to diversity* (2011 M2c and 2015 M2c), to test our hypotheses about the differential hypothesized relationships of *perceived climate for inclusion of minorities* among the majority and minority groups. If the hypotheses hold, the model that allows for differential relationships between the majority and minority group should lead to an improved fit compared to 2011 M1 and 2015 M1 models.

In the final stage of our analysis approach we examined if the hypothesized relationship of *perceived climate for inclusion of minorities* is robust and not incidental. To this end we examined if the model results for 2011 and 2015 were similar in direction and size thereby providing evidence for a systematic difference between majority and minority groups. This was done by comparing the fit of three nested models and evaluating the best fit by varying the restrictions of the structural paths. In model M3_1 we assume that hypothesized relationships are similar across both groups and time; model M3_2 assumes that the hypothesized relationships between the constructs are equal across time, but may differ for majority and minority groups; and model M3_3 assumes that hypothesized relationships differ across both groups and time. The measurement part of all three models was constrained to be measurement invariant for all three models.

### Fit indices

In order to test the models (M0 – M3) we use formal chi-square tests as well as three often used fit indices: the root mean square error of approximation (RMSEA; Steiger, 1989), the Tucker-Lewis index (TLI; Tucker and Lewis, 1973) and the comparative fit index (CFI; Bentler, 1990). The RMSEA is an absolute fit index that examines closeness of fit of the model. A RMSEA value of more than 0.1 is seen as an indication of poor fit, a value of 0.05 to 0.08 as acceptable and a value below 0.05 as good to very good (Hu and Bentler, 1999). The TLI and CFI compare the fit of the model under consideration with the fit of the baseline-model (here the M1 model). Fit is considered adequate if the TLI and CFI values are above 0.90, better if they are above 0.95.

## Results

### Preparatory stage: measurement equivalence

In the base model (M0) we performed a multi-group confirmatory factor analysis (MGCFA) to test whether the constructs have the same meaning for minority and majority groups. The invariant measurement model fits both the 2011 and the 2015 sample well: 2011 M0 (RMSEA = 0.039, CFI = 0.973, TLI = 0.975) and 2015 M0 (RMSEA = 0.049, CFI = 0.973, TLI = 0.977). This allows for a meaningful comparison of the latent scores between the majority and the minority groups in both 2011 and 2015. Supplementary Table S2 provides the measurement coefficients (factor loadings) of the (latent) constructs in the 2011 and 2015 measurement invariant model.

### Results for differential relationships between majority and minority groups

To test our hypotheses regarding different associations of perceived inclusive climate among the majority and minority groups, a set of models was specified: a model that constrained all structural paths to intergroup equivalence (M1: same associations exist between latent constructs across groups) and models that released the structural paths for native Dutch from *Perceived climate for inclusion of minorities* to the dependent variables (M2 models: associations between latent constructs differ across groups).^8^ This was done for both the 2011 sample and the 2015 sample. Table 2 presents chi-squares and fit measures for these models for both 2011 and 2015.

**Table 2:** Chi-squares and fit measures for the structural models: invariant structural model and partial invariant models.

Sample	Model	Fit indices					
		Chi-square (df)	*N* ^1^	RMSEA (90% CI)	CFI	TLI	Formal Chi-square test against M1^2^
2011	M1	1066.851 (93)	6,815	0.055 (0.052–0.058)	0.932	0.934	-
	M2a	934.087 (92)	6,815	0.052 (0.049–0.055)	0.941	0.943	*Χ*^2^_(1)_ = 140.896, *p* < 0.000
	M2b	823.341 (92)	6,815	0.048 (0.045–0.051)	0.949	0.950	*Χ*^2^_(1)_ = 159.945, *p* < 0.000
	M2c	899.480 (92)	6,815	0.051 (0.048–0.054)	0.944	0.945	*Χ*^2^_(1)_ = 145.678, *p* < 0.000
2015	M1	966.161 (93)	5,069	0.061 (0.057–0.064)	0.953	0.955	-
	M2a	699.053 (92)	5,069	0.051 (0.048–0.055)	0.967	0.968	*Χ*^2^_(1)_ = 223.681, *p* < 0.000
	M2b	850.690 (92)	5,069	0.057 (0.054–0.061)	0.959	0.960	*Χ*^2^_(1)_ = 80.741, *p* < 0.000
	M2c	1097.874 (92)	5,069	0.066 (0.062–0.069)	0.946	0.947	*Χ*^2^_(1)_ = 18.997, *p* < 0.000

M1: model with structural paths between constructs constrained to be equal for the majority and minority groups. M2a: model with path from Perceived climate for inclusion of minorities to ethnocentrism released. M2b: model with path from Perceived climate for inclusion of minorities to reluctance to engage in interethnic contact released. M2c: model with path from Perceived climate for inclusion of minorities to resistance to diversity released. ^1^For the 2011 analysis there were 8 cases with missing information on all variables. For the 2015 analysis there were 6 cases with missing information on all variables. These were excluded from the analysis. ^2^In this test the chi-square difference can be smaller or larger than the observed difference between the tested models. This is due to the Santorra Bentler correction.

The formal chi-square test, as well as the improvement in fit measures, indicated that, as hypothesized, perception of an inclusive environment has different implications for intergroup attitudes for the majority than for the minority group when we consider *ethnocentrism* and *reluctance to engage in interethnic contact*. *Climate for inclusion* is positively associated with *ethnocentrism* in the majority group, meaning that when the majority group perceives a climate that is more open and just towards minority groups, they have a higher preference for their ethnic group compared to other ethnic groups. In contrast, in minority groups, climate for inclusion is negatively associated with *ethnocentrism*, meaning that when these groups perceive that the societal climate is more open and accepting of minorities their relative preference of their own ethnic group compared to the majority group is smaller (M2a). Table 3 shows the coefficients of the released paths for both majority and minority group.

**Table 3:** Coefficients and standard errors of the released paths for both majority and minority group.

	Effect of climate for inclusion on:	Majority estimate (SE)	Minority estimate (SE)	Model
2011	Ethnocentrism	0.136 (0.016)*	−0.154 (0.017) *	2011_M2a
	Reluctance to engage in interethnic contact	0.489 (0.056) *	−0.233 (0.042) *	2011_M2b
	Resistance to diversity	0.799 (0.052) *	0.248 (0.028) *	2011_M2c
2015	Ethnocentrism	0.143 (0.019) *	−0.216 (0.016) *	2015_M2a
	Reluctance to engage in interethnic contact	0.367 (0.046) *	−0.204 (0.055) *	2015_M2b
	Resistance to diversity	0.533 (0.034) *	0.268 (0.055) *	2015_M2c

*p < 0.01.

Also, in line with our hypothesis we find that *climate for inclusion* of minorities is positively associated with *reluctance to engage in interethnic contact* among the majority group. For minority groups, the effect is opposite as hypothesized: perceiving a more inclusive climate goes hand in hand with less *reluctance to engage in interethnic contact* with the majority group. This is true for both the 2011 and the 2015 sample (M2b).

In line with our reasoning, the effects of *climate for inclusion* of minorities on *ethnocentrism* and *reluctance to engage in interethnic contact* run in opposite directions for majority and minority groups. However, even though we expected the same differential pattern for *resistance to diversity*, this is not what we find in model M2c for 2015. This model did show a small but statistically significant decrease in fit as compared to the model restricting this to be equal between the majority and minority group. Freeing that parameter does not improve the overall fit of the model. This means that in the 2015 study, the relationship between inclusive environment perceptions and resistance to diversity was similar for both the majority and minority groups. To the extent minority *and* majority groups perceive a more positive inclusion climate, they both tend to resist diversity more. Thus, even though H1a is supported, *Hypothesis 1b* is supported for ethnocentrism and reluctance to engage in interethnic contact but not for resistance to diversity.

In the 2011 sample, the direction of the association between *climate for inclusion* and *resistance to diversity* is the same for majority and minority groups. However, the strength of the relationship between the concepts is stronger for the majority group than for the minority group.^9^ Taken together, these findings support *Hypothesis 1a* and partially support *Hypothesis 1b*. We discuss possible reasons and implications in the discussion section.

### Robustness of the relationships

Finally, we present the results of the three nested M3 models (M3_1 – M3_3) with varying restrictions for the structural paths between the same constructs across the different groups between both samples to ensure the robustness of the relationships. Table 4 presents chi-squares and fit measures for the models, testing if relationships are similar across both groups and samples (M3_1), if relationships are equal across samples, but may differ for majority and minority groups (M3_2), and if relationships differ across both groups and samples (M3_3). This allows for a formal test of the hypothesis that the differential relationships are the same in size and directions across samples (M3_2) thereby providing evidence for a systematic difference between majority and minority groups.

**Table 4:** Fit indices and chi-square test for models testing robustness across samples.

Model	Fit indices					
	Chi-square (*df*)	*N* ^1^	RMSEA (90% CI)	CFI	TLI	Formal Chi-square test^2^
M3_1	2063.681 (219)	11,884	0.053 (0.051–0.055)	0.943	0.953	-
M3_2	1078.497 (216)	11,884	0.037 (0.035–0.039)	0.973	0.978	M3_1: *Χ*^2^_(3)_ = 606.694, *p* < 0.000
M3_3	1221.438 (210)	11,884	0.040 (0.038–0.042)	0.965	0.973	M3_2: Χ^2^_(6)_ = 18.922, *p* < 0.004

^1^For the analysis there were 14 cases with missing information on all variables, 8 for the 2011 sample and 6 from the 2015 sample. These were excluded from the analysis. ^2^In this test the Chi-square difference can be smaller or larger than the observed difference between the tested models. This is due to the Santorra Bentler correction.

The results from Table 4 indicate that the model testing whether the structural paths between the constructs are equal across both samples, but vary between the majority and minority groups has the best fit (M3_2) and clearly fits the data structure well, thereby providing support for our hypotheses.

Table 5 shows the coefficients of the structural paths for both majority and minority groups fitted to be equal across samples but different between groups. The factor loadings of this model (M3_2) can be found in the Supplementary Table S2. The table shows opposite associations for majority and minority groups for perceived *climate for inclusion* of minorities on both *ethnocentrism* and *reluctance to engage in interethnic contact*. Whereas the perception of an inclusionary climate for minorities is associated with more ethnocentrism in the majority group, it is associated with less ethnocentrism among minority groups. Similarly, the perception of an open and inclusive climate is associated with less negative attitudes towards interethnic contact among minority groups, whereas it is associated with greater objection to interethnic contact in the majority group. Finally, we found that the majority group’s perception of an inclusive climate for minorities is linked to their resistance towards a culturally diverse society and negative attitudes towards ethnically diverse neighborhoods. Interestingly, we find a similar pattern for minority groups, but to a significantly lesser extent. As shown by the formal test, this pattern applies to both the 2011 and the 2015 sample. Taken together, the consistent findings indicate a stable pattern in which a perception of a more inclusive, diverse and equal society has largely different implications for majority and minority groups.

**Table 5:** Structural path coefficients and standard errors of final model (different between groups and consistent across samples).

Perceived climate for inclusion of minorities on	Majority group coefficient (SE)	Minority groups coefficient (SE)
Ethnocentrism	0.152* (0.016)	−0.170* (0.012)
Reluctance to engage in interethnic contact	0.437* (0.047)	−0.232* (0.037)
Resistance to diversity	0.661* (0.036)	0.273* (0.028)

*p < 0.01.

## Discussion

This study investigated the paradoxical effects of perceived national inclusion climate for minorities on both majority and minority groups in the Netherlands. Our results show that a perception of an inclusive climate is associated with positive attitudes towards interethnic relations in minority groups, but has opposite effects on the majority group. In particular, the perception of a commitment to equal opportunities and openness toward ethnic minorities is positively correlated with ethnocentric attitudes and a hesitancy to engage in interethnic contact within the majority group. Additionally, this perception is positively associated with opposition to ethnic diversity in general, including perceptions that the mere presence of ethnic minorities has a negative impact on neighborhoods.

This work highlights a paradox that arises when nations strive to create a more equitable and just society. Our findings indicate that when efforts to create a level playing field are perceived as successful, they can paradoxically lead to social tensions because minorities’ intentions to engage with the majority in these environments may be met by rejection and distance from the members of the majority group. An additional, unexpected, finding further suggests that this paradox can extend to some parts of intraminority relationships: perceived national inclusion climate for minorities is negatively associated with openness to increased ethnic diveristy in society and neighborhoods among minorities in our sample. We discuss the implications below.

### Theoretical contributions

The findings for the majority group generally support the basic propositions of the instrumental model of group conflict (Esses et al., 1998, 2005). Because an environment that signals social mobility and opportunity richness for minority groups can be construed as a threat to the economic and cultural hegemony of the majority group, perceptions of an inclusionary climate may motivate the majority group to suppress the source of competition they experience. One way to do this is to report negative attitudes towards and to avoid the competitive minority groups. The current work sheds light onto the different forms such coping mechanisms can take and suggests that it ranges from unfavorable intra-personal affective responses -ethnocentric views- to a rejection of direct contact with minorities to opposition to diversity in general.

In contrast with the majority group’s avoidant responses, we find that minority groups’ perceptions of an inclusionary climate are associated with greater inclination toward engagement. In these environments, minorities report more favorable intra-personal affective responses (i.e., less ethnocentrism) and are more positive with regards to direct contact with the majority group. These findings build on the instrumental model of group conflict by showing a potential boundary condition: while environments that signal social mobility may be linked with avoidant tendencies among high status groups, for low status groups the opposite may be true.

Intriguingly, contrary to what we expected, we found a positive link between the perception of an inclusive climate and resistance to ethnically diverse settings, even within minority groups. This association is stronger for majority members than for minority groups, but – in contrast to our other findings – the direction of the association for both groups is the same. This finding suggests that ethnic minority groups, much like the majority group, may associate an increasing presence of (other) minorities as a threat in an inclusive climate for minorities. That is, the perception of an inclusive climate may also contribute to the development of fixed pie perceptions within intraminority relationships, contributing to specific outgroup distancing intentions between minority populations. This finding is critical as it (a) uniquely shows the relevance of instrumental model of group conflict (Esses et al., 1998, 2005) for intraminority relationships, and in so doing (b) moves beyond simplistic in- versus out-group dynamics. By examining specific minority populations and their intergroup dynamics within a contextualized framework, our findings hold significance for advancing the development of theories that explain the emergence of intergroup threats beyond majority-minority relationships.

It is noteworthy that the intragroup findings in the present research may also reflect (a fear for) enhanced competition for resources and services in increasingly mixed neighborhoods, that are often segregated along ethnic lines. For instance, in the Netherlands, there is a significant shortage of qualified school teachers, particularly in economically disadvantaged urban neighborhoods. Given that ethnic minorities tend to reside in these areas more frequently, these shortages have a disproportionate impact on the quality of schools and the provided education for their children. Resistance to diverse neighborhoods for minority groups then may also reflect concerns for the quality of services and living conditions in one’s neighborhood. Taken together, the findings among the minority groups show that perceptions of minority inclusion, where minorities have a chance to ascend in the hierarchical layers, are positively related to minorities’ attitudes towards the majority group but -at least to some extent- negatively towards the presence and increased share of (other) minority groups.

### Limitations and future directions

To fully grasp the scope and implications of our current research, it is important to consider its contextual relevance and boundaries. As previously discussed, we leveraged data from two waves of a large, nationally representative, repeated, cross-sectional survey, which offers several advantages. These strengths include our ability to construct and test a theoretical model that uniquely examines the perspectives and psychological dynamics of minority groups. Furthermore, the data allowed us to replicate our initial findings using a separate survey conducted with a significant time gap. Consequently, this approach enhances our confidence in the established relationships between variables and mitigates to a large degree the risk of Type 1 errors often associated with cross-sectional research reliant on a single data collection instance.

Nonetheless, it is important to acknowledge the limitations stemming from the cross-sectional nature of our datasets and our reliance on self-reported individual-level perceptions. While we present and test a theoretical model that places perceived inclusion before outcome measures like ethnocentrism and interethnic contact, we recognize that these data do not allow us to determine causation. In other words, we cannot rule out the possibility that individuals scoring higher in ethnocentrism and/or showing reluctance toward interethnic contact might also perceive a highly inclusive national climate toward ethnic minorities. Experimental approaches or time-lagged designs are necessary to establish the direction of these relationships more robustly.

Future research may also benefit from incorporating additional key variables to enhance our understanding of potential reverse causality. One such variable is individual differences in system justifying beliefs, the extent to which individuals have internalized the legitimacy of existing social arrangements (Jost, 2019). For example, the current data reveal a positive association between ethnocentrism as well as the rejection of intergroup contact, and the perceived inclusion of minorities (i.e., the perception of an environment that offers minority groups equal opportunities) among members of the majority group. Whether these positive relationships are linked to the legitimization of inequality remains an unanswered question. Recent studies show that among the members of the majority group system justifying beliefs are negatively associated with perceived inequal treatment of minorities (Bahamondes et al., 2019; Suppes et al., 2019), positively associated with group-based discrimination (Bahamondes et al., 2020), and seeing progress among minorities as threat to one’s own group’s status (Wilkins and Kaiser, 2014). Recent research thus underscores the significance of considering system justification as a crucial variable for a more comprehensive understanding of the concepts and relationships examined in this study. By taking this variable into explicit consideration, future work can enhance our understanding of potential reverse causality and its underlying factors.

Our research provides insights into the relationships between the majority group’s perceptions of inclusive climate for minorities and negative attitudes toward minorities. However, it does not elucidate the accuracy or exact origin of these perceptions (and the concomitant threat). At least two critical questions thus remain. The first question is: to what degree does this perceived threat accurately reflect the real economic conditions of the majority group in relation to the improving situation of minority groups? An examination of the statistics suggests that the economic position of minorities in the period between 2003 and 2015 has been consistently behind the majority group, as evident from significantly higher levels of unemployment, lower salaries and lower chance of having permanent jobs (Huijnk and Andriessen, 2016). This suggests that the perceived economic threat in our study may not be an accurate reflection of an economic reality, at least at the group level.

The second question is: where do these seemingly inaccurate perceptions come from? To what extent does this perceived threat arise in response to pro-diversity policies and actions, as opposed to being influenced by a broader discourse encompassing media coverage and anecdotal evidence? We speculate that they arise as a response to both policies and programs targeting minority inclusion and participation and the broader discourse including media coverage and political narratives. Research suggests that non-target group members, such as the majority group, often perceive pro-diversity policies and actions as signals of unfair disadvantage to their group (Brannon et al., 2018; Brown and Jacoby-Senghor, 2021). It is also not surprising that public discourse on the “deteriorating position of the majority group” is rich in examples where these policies are construed in ways consistent with this worldview. For instance, the article quoted at the beginning discusses the majority group’s discontent seemingly arising from government policies aimed at promoting the participation of minority groups through support for multicultural community centers (Elsevier Weekblad, 2016). Further, governmental policies prioritizing refugee families in public housing allocation are seen as unfairly taking resources away from the members of the majority group, a viewpoint that has received ample attention in the narratives of right-wing political parties (NOS.nl, 2023), which arguably adds to the discontent felt among a portion of the citizens.

Given our focus on *perceived* inclusion climate and the associated threat, the accuracy and origins of the information used by respondents to provide an answer to the predictor variable in our model are outside the scope of this study. Nonetheless, better understanding these factors is important for both advancing theory and developing effective interventions. Future research could explore how the perceived sense of threat among majority group members arises, examining the contributions of governmental policies, programs, and societal discourse.

## Conclusion

This study highlights the paradoxical effects of creating an inclusive environment for ethnic minorities in European societies based on two waves of a repeated, nationally representative, cross-sectional survey, totalling over eleven thousand respondents. While an inclusive environment may improve social mobility and opportunities for minority groups, it may also result in negative intergroup sentiments and attitudes towards immigrants among the majority group. Conversely, an inclusive environment may foster more positive intergroup attitudes and affect for minority groups. Governments and other institutions should remain cognizant of these dynamics and work towards inclusion efforts that alleviate zero-sum perceptions of progress among different groups. This can be achieved through clearer articulation of goals and benefits for the broader society in the long term, as well as through comprehensive public campaigns to correct misinformation.

## Data availability statement

Publicly available datasets were analyzed in this study. This data can be found at: doi.org/10.17026/dans-xfv-2vx4 and doi.org/10.17026/dans-xep-by9x.

## Ethics statement

Ethical approval was not required given the analysis of publicly available data sets that are not collected by the authors. CBS (Statistics Netherlands - SN) was responsible for selecting the sample and providing the fieldwork agency with the contacting details of the sampled persons (which includes but is not limited to a DPA and secure transfer of information). The studies were conducted in accordance with the local legislation and institutional requirements. Written informed consent for participation was not required from the participants or the participants’ legal guardians/next of kin in accordance with the national legislation and institutional requirements relevant for the data collecting agency.

## Author contributions

IA and SG formulated the research question and hypotheses. IA, SG, JK, and AH contributed to the conceptualization of the manuscript, the data on which this manuscript is based was originally collected for a project by the Netherlands Institute for Social Research, funded by the Dutch Ministry of Social Affairs and Employment, Integration office, and interpreted the findings. JK conducted the statistical analyses. IA, SG, and JK wrote the first draft of the manuscript. AH provided critical revisions. All authors reviewed and provided critical revisions to the final draft of the manuscript.

## Footnotes

^1^StatLine - Bevolking; migratieachtergrond, generatie, lft, regio, 1 January; 2010–2022 (cbs.nl)

^2^CBS uses the following official definition to describe a non-Western person in the Netherlands: “Every person residing in the Netherlands of whom one or both parents were born in Africa, Latin America, Asia (excluding Indonesia and Japan) or Turkey.”

^3^Data for the SIM 2011 survey was gathered in the years 2010 and 2011 (from November 2010 to June 2011). For sake of brievity we call it the SIM 2011 survey. At the time of our analyses these two waves were the most recent, publicly available data sets.

^4^2011 fieldwork was conducted by Gfk and Labyrinth. 2015 fieldwork was conducted by TNS Nipo [now Kantar Public] and Labyrinth.

^5^ https://www.cbs.nl/nl-nl/faq/specifiek/wat-verstaat-het-cbs-onder-een-allochtoon

^6^Three factors (*perceived climate for inclusion, reluctance to engage in interethnic contact, resistance to diversity*) have ordered categorical indicators and therefore the WLSMV (Mean- and Variance-adjusted Weighted Least Square) estimator has been used to address the multivariate normality assumption (Lubke and Muthén, 2004). To reflect the data structure, a cluster variable was included to allow for a correction of possible interviewer-dependent correlation between the answers of respondents that were interviewed by the same interviewer. A weighting variable was also included to correct for potential nonresponse error and unequal inclusion probabilities.

^7^It is important to note that the M0 models do not contain the directly observed measure of ethnocentrism. As a result of the addition of the measure of ethnocentrism in the M1 model, the M1 models are expanded and thus not nested within the M0 models. This poses no problems as no formal tests will be conducted between M0 and M1 because this is not relevant for our study.

^8^In both models, measurement models were constrained to invariance. See Online Supplement for factor loadings.

^9^We add, however, that the difference in sample size between 2010/2011 and 2015 (about 1800 cases) may account for the difference in findings between the two samples. Possibly the difference in strength between the majority and minority group in the 2015 sample would have reached statistical significance (as indicated by a drop in fit and formal chi square test) with an equally large sample size.
