# Supplementary material for: Examining the divergent effects of perceived inclusion of ethnic minorities on majority and minority groups’ inter-ethnic responses
Source: Front Psychol. 2023 Nov 8;14:1242595. doi: 10.3389/fpsyg.2023.1242595 (PMC10663700; doi:10.3389/fpsyg.2023.1242595)
Supplement: Supplementary file 1 [file Table_1.pdf]

Online Supplement

Table S1: Overview of labels

Table S2: Factor loadings for latent constructs in invariant condition separately for 2011 and 2015

Figure S1: Tested structural model

Table S1

Overview of labels

Construct	Item	Label in dataset	Label used in paper
Perceived climate for inclusion			
	The Netherlands is a hospitable country for ethnic minorities.	NLGASTV	hosp
	The Netherlands is open to foreign cultures.	OPENCULTA	open
	Ethnic minorities have every chance in the Netherlands.	KANSALLO	chance
	In the Netherlands the rights of ethnic minorities are respected.	RESPRECH	chance
Ethnocentrism			
	'Feeling' thermometer towards individuals with a native Dutch background.	TEMPAUT	tempnatd
	'Feeling' thermometer towards individuals with a Turkish background.	TEMPTUR	temptur
	'Feeling' thermometer towards individuals with a Moroccan background.	TEMPMAR	tempmor
	'Feeling' thermometer towards individuals with a Surinamese background.	TEMPSUR	tempsur
	'Feeling' thermometer towards individuals with a Dutch-Caribbean background.	TEMPANT	tempant
Resistance to diversity			
	Too many ethnic minorities live in the Netherlands.	VEELALLO	many

	Neighborhoods deteriorate when too many ethnic minorities live there.	SLECHTWK	deter
	It is a good thing when a society consists of different cultures. (r)	CULTDIV	cultdiv
Reluctance to engage in interethnic contact			
	How much would you object to one of your children having many friends from ethnic minority groups [native-Dutch participants]/ many native Dutch friends [ethnic minority participants]?	AFKVRIKI	friends
	How much would you object to one of your children choosing a partner from an ethnic minority group[native-Dutch participants]/ a native Dutch partner [ethnic minority participants]?	AFKPARKI	partner

Table S2

Factor loadings for latent constructs in invariant condition separately for 2011 and 2015 (M0) and across 2011 and 2015 (M3_2)

	2010_M0	2015_M0	M3_2
Climate for inclusion			
- hosp	1.0	1.0	1.0
- open	0.702	0.757	0.696
- chance	0.860	0.847	0.913
- rights	0.698	0.888	0.774
Resistance to diversity			
- many	1.0	1.0	1.0
- deter	0.553	0.730	0.613
- cultdiv	0.358	0.490	0.406
Reluctance of engage in interethnic contact			
- partner	1.0	1.0	1.0
- friends	0.813	0.700	0.651

Figure S1

Tested structural model

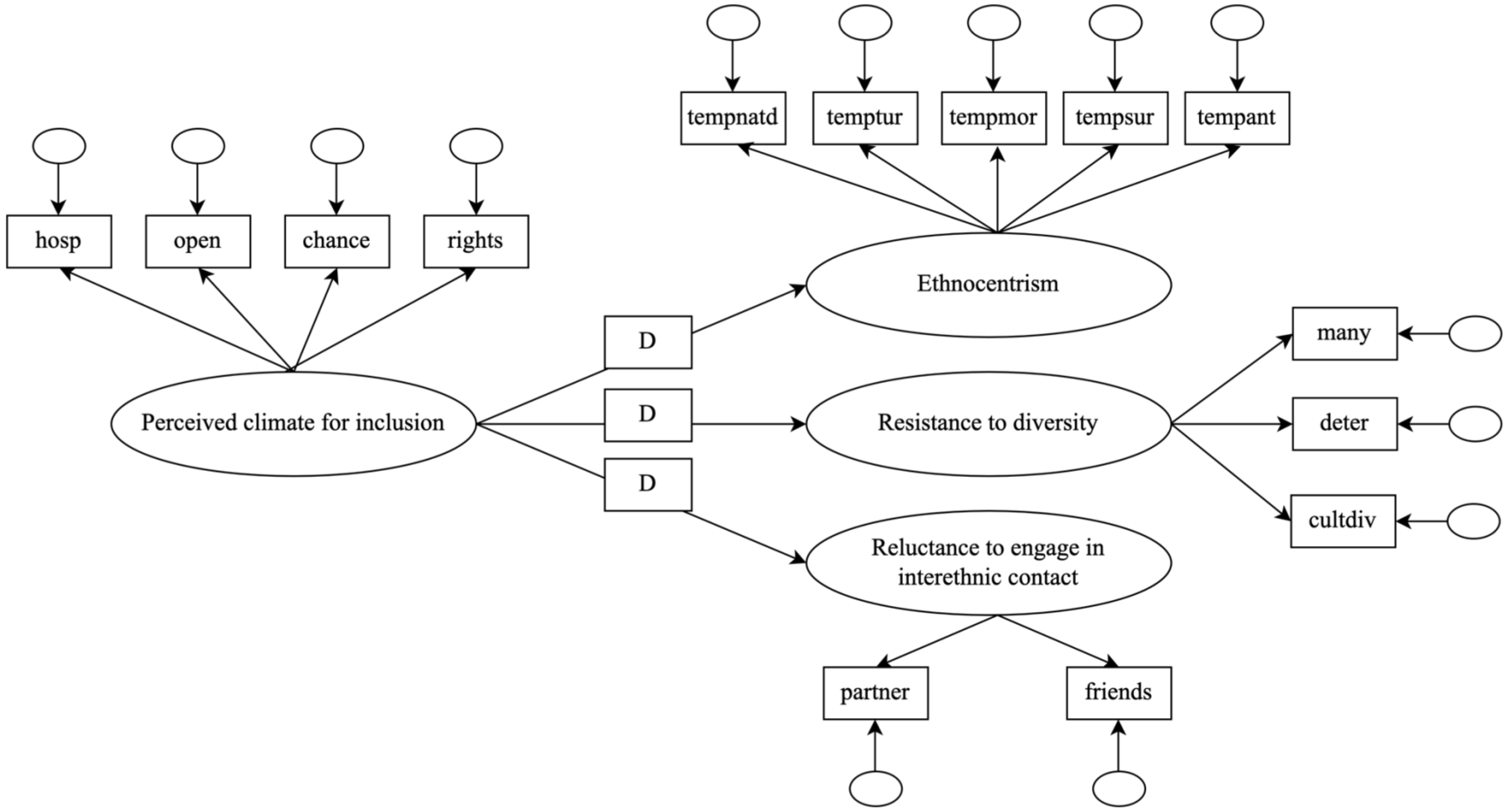

Note. D stands for different structural path for majority and minority groups.
